# An external peer review instrument for Korean Medicine clinical practice guideline drafts (KM-EPRI): development and content validation

**DOI:** 10.3389/fmed.2026.1926917

**Published:** 2026-09-23

**Authors:** Hee Jung Lee, Yoon Jae Lee, Hyo-Weon Suh, Keumji Kim, Jihyun Sang, Junhyeok Yi, Seungwon Shin

**Affiliations:** 1 Department of Korean Medicine, College of Korean Medicine, Daegu Haany University, Gyeongsan, Republic of Korea; 2 Jaseng Spine and Joint Research Institute, Jaseng Medical Foundation, Seoul, Republic of Korea; 3 Clinical Evidence Research Team, Division of Healthcare Research, National Evidence-Based Healthcare Collaborating Agency, Seoul, Republic of Korea; 4 Korean Medicine Clinical Practice Guidelines Team, National Institute for Korean Medicine Development, Seoul, Republic of Korea; 5 College of Korean Medicine, Sangji University, Wonju, Gangwon, Republic of Korea; 6 National Agency for Korean Medicine Innovative Technologies Development, National Institute for Korean Medicine Development, Seoul, Republic of Korea

**Keywords:** clinical practice guidelines, Delphi technique, directed qualitative content analysis, external peer review, instrument development and content validation, traditional Korean medicine

## Abstract

**Background:**

Korean Medicine clinical practice guidelines (KM-CPGs) undergo mandatory external peer review of their drafts during development, yet reviewers lack a standardized, validated instrument to guide this task. Widely used appraisal tools assess the quality of completed guidelines rather than support the section-level review of a draft. We aimed to develop an external peer review instrument for KM-CPG drafts (KM-EPRI) and to assess its content validity.

**Methods:**

In this two-phase methodological study, a draft instrument was first derived by directed qualitative content analysis of the external review comments produced for previously published KM-CPGs, with each comment coded into one of four predefined review modules: the clinical questions (CQ), the introductory components (CPG-I), the development methods (CPG-M), and the recommendations (CPG-R). The content validity and acceptability of the items were then assessed through a modified Delphi study with a multidisciplinary panel of KM clinicians and evidence-based medicine methodologists. Consensus was defined a priori as an item-level content validity index (I-CVI) ≥0.80. The scale-level content validity index (S-CVI/Ave), Kendall’s coefficient of concordance (W), and the interquartile range (IQR) were reported as supporting measures.

**Results:**

From 3,630 external review comments, 2,073 content-related comments were consolidated and refined into a finalized instrument of 117 review items across 4 modules and 33 subdomains. All 10 invited experts participated (response rate 100%, no attrition), and every item reached the consensus threshold in a single round under a pre-defined early-stopping rule. All 117 items had an I-CVI ≥0.80, the S-CVI/Ave was 0.976, and the IQR was ≤1 for every item (median 0.75). Kendall’s W was low (0.196), reflecting a ceiling effect from uniformly high ratings rather than disagreement among panelists.

**Conclusion:**

KM-EPRI can serve as a consensus-based, structured reference for the external peer review of KM-CPG drafts during development. Its effect on review quality and inter-rater reliability remains to be evaluated in practice.

## 1 Introduction

Clinical practice guidelines (CPGs) translate the best available evidence into actionable recommendations. Their trustworthiness depends not only on rigorous development but also on independent appraisal before dissemination (1). Guideline-producing bodies therefore embed external review or stakeholder consultation as a defined step in their development processes. The U.S. National Academy of Medicine (formerly the Institute of Medicine) includes external review among its standards for trustworthy guidelines, specifying who should review a draft, how comments are documented, and when drafts are opened to public comment (1). The Guidelines International Network likewise identifies guideline review as one of its core components of high-quality guidelines (2). The methods manuals of the National Institute for Health and Care Excellence (3), the Scottish Intercollegiate Guidelines Network (4), the World Health Organization (5), the National Health and Medical Research Council (6), and the U.S. Preventive Services Task Force (7) each require consultation, peer review, or independent expert and methodological review of draft guidelines.

In the Republic of Korea, Korean Medicine (KM) CPGs (KM-CPGs) are developed by multidisciplinary groups that apply Cochrane methods for systematic review together with the Grading of Recommendations Assessment, Development and Evaluation (GRADE) approach (8), consistent with national methodological standards for CPG development (9, 10). A new guideline is developed de novo on this basis, whereas an update follows a hybrid approach that combines adopting or adapting existing recommendations with de novo development where no suitable source recommendation exists. Each guideline is organized into standardized components following the KM-CPG development manual (11). Since the launch of a government-funded program in 2020, the number of KM-CPGs has grown steadily; more than 50 are now registered in the national clearinghouse for KM (12). During development, draft guidelines undergo mandatory external peer review to safeguard scientific validity and clinical acceptability. This review is coordinated by the national KM-CPG center operated by the National Institute for Korean Medicine Development. The reviewers are experts in guideline methodology and in the relevant clinical field, and they must not be members of the clinical practice guideline development group (CDG). They examine the guideline at stages from the planning of the key clinical questions through to the final draft (11). The completed guideline then undergoes a multistage accreditation process. During accreditation, it is appraised using the Appraisal of Guidelines for Research and Evaluation II (AGREE II) instrument (11, 13).

Despite this procedural infrastructure, the guidance that reviewers apply when examining a guideline draft remains largely unstandardized. AGREE II, the most widely used appraisal tool, was designed to assess the overall quality of a completed guideline rather than to guide the section-level review of a draft. Within its items, external review appears only as a single item confirming whether the guideline was externally reviewed before publication. It does not specify how that review should be conducted (13). To the best of our knowledge, structured, publicly available tools that reviewers use to appraise guideline drafts are likewise not described in the methods manuals of guideline organizations, which generally rely on open-ended comment or unstructured consultation formats (3, 7).

The absence of an explicit, shared review instrument has tangible effects. Without common reference points, reviewers’ judgments are difficult to standardize, and the clarity of the underlying tool is an important determinant of inter-rater reliability (14). Expectations may also diverge between developers and reviewers: even guidelines judged to be of high technical quality by the original AGREE instrument may be regarded by expert panels as insufficiently comprehensive or valid (15). In practice, an unstandardized review process may further reduce consistency and efficiency. Together, these problems indicate the need for a standardized, validated instrument that reviewers can refer to when examining KM-CPG drafts.

Against this background, we conducted a two-phase study to develop and validate an external peer review instrument for KM-CPG drafts (KM-EPRI), as distinct from the post-hoc appraisal of completed guidelines such as AGREE II. In the development phase, we derived a draft instrument from prior external review comments through directed qualitative content analysis. In the validation phase, we examined its content validity and acceptability through a modified Delphi study with a multidisciplinary expert panel. Our aim was to produce a consensus-based instrument with established content validity to support external peer review of KM-CPG drafts.

## 2 Materials and methods

### 2.1 Study design and instrument framework

We conducted a two-phase methodological study to develop and validate an external peer review instrument for KM-CPG drafts. In Phase A (development), the items of the instrument were derived by a directed qualitative content analysis of the external review comments that independent expert reviewers had actually produced during the development of previously published KM-CPGs. In Phase B (validation), the content validity and acceptability of these items were assessed through a modified Delphi study.

We defined four review modules, each corresponding to a distinct component of a KM-CPG (11). The CQ module addresses the key clinical questions, formulated in the Population–Intervention–Comparison–Outcome (PICO) format, specified at the planning stage. The CPG-I module concerns the introductory and supporting components of the draft guideline: the summary, introduction, disease overview, guideline utilization, clinical pathway diagram, and dissemination tools. The CPG-M module applies to the development-methods section, covering the systematic review and meta-analysis process and the derivation of the level of evidence and strength of recommendation under GRADE. The CPG-R module pertains to the recommendation section, which presents the summaries of the included evidence, the meta-analytic and qualitative synthesis results, and the resulting GRADE level of evidence and strength of recommendation.

### 2.2 Phase A: development of the draft instrument

The instrument was developed from the source comments in five steps: source-data collection, editorial screening, classification and consolidation, finalization, and meta-evaluation item integration. The classification framework, comprising the four review modules, was defined a priori. Three researchers carried out each step collaboratively, coding the comments against this predefined framework and reconciling their judgments through discussion. An assignment was adopted only when the three reached consensus.

#### 2.2.1 Source-data collection (Step 1)

The source guidelines were all thirteen KM-CPGs that had been developed in accordance with the official KM-CPG development manual, version 2.0 (published 30 June 2022), and had completed publication through external peer review and the official accreditation process by the end of August 2025 (11, 12). Version 2.0 revised the components a guideline must contain and the procedure for external peer review, so only guidelines developed under it were used as source data. From these, all comments submitted by independent external reviewers to the CDGs were collated, irrespective of reviewing body or review round. The number of external reviewers and review rounds for each source guideline is provided in Supplementary Table S1.

#### 2.2.2 Editorial screening (Step 2)

Comments reflecting editorial or formatting issues (typographical errors, notation, reference formatting, and duplicated or omitted text) were removed, leaving the content-related comments.

#### 2.2.3 Classification and consolidation (Step 3)

Because the components of a KM-CPG are predefined by an established development framework, we analyzed the content-related comments using a directed qualitative content analysis: the review-module framework served as the initial categorization matrix into which each comment was coded, rather than allowing categories to emerge from the data (16, 17). Each comment was assigned to the CQ module for the key clinical questions reviewed at the planning stage, or to the CPG-I, CPG-M, and CPG-R modules for the respective parts of the draft guideline. Within each module, recurring comments addressing the same point were consolidated into module-level draft items, so that each item captured a generalizable review point rather than a guideline-specific remark. Because the modules map onto guideline sections whose content overlaps, and because each module is intended to stand alone for independent review, consolidation was performed within modules rather than across them. A review point relevant to several sections was retained in each corresponding module instead of being merged into a single cross-module item. The resulting recurrence preserves the self-sufficiency of each module and supports explicit checks of consistency between sections.

#### 2.2.4 Finalization (Step 4)

Items not amenable to expert content-validity review were then removed or merged. These were redundant items and value-matching items (the latter checking whether the analytic or outcome values recurring in several parts of a guideline are mutually consistent), whose adequacy is established by direct verification rather than by expert consensus. The finalized items were organized into subdomains that follow the section structure prescribed by the KM-CPG development manual, so that each item maps to the corresponding part of a draft guideline.

#### 2.2.5 Meta-evaluation item integration (Step 5)

Alongside the finalized instrument, one module-system meta-evaluation item was added to the set assessed in Phase B. This item states that the review-module system (CQ, CPG-I, CPG-M, CPG-R) is appropriately structured to review a guideline’s quality and clinical applicability from multiple perspectives. Because it evaluates the module framework as a whole rather than any individual review point, it was assessed together with the instrument in the modified Delphi but is not a component of the instrument.

### 2.3 Phase B: modified Delphi validation

Phase B validated the draft instrument through a modified Delphi study. The modified Delphi was used to assess the content validity and acceptability of the items and to establish consensus (18, 19). The study was approved by the Institutional Review Board of Sangji University (Wonju, Republic of Korea; approval No. 1040782-260311-HR-13-188; approved 29 April 2026). All participants provided written informed consent, and their responses were anonymized. Phase B was conducted and reported in accordance with the standardized reporting guideline for Delphi studies (DELPHISTAR) (20). Methodological and statistical aspects were addressed within the author team, which included evidence-based medicine (EBM) methodologists.

#### 2.3.1 Expert panel and recruitment

The panel was multidisciplinary, comprising KM clinicians and EBM methodologists. Eligible participants were adults aged ≥19 years who met at least one of the following: (i) prior participation in the planning, development, review, and/or accreditation of a KM-CPG; (ii) EBM methodological expertise (e.g., systematic review, GRADE); or (iii) ≥5 years of KM clinical experience. Experts were identified by purposive sampling with snowball referral (21) and invited by email with study information. Patient or public representatives were not included, as the instrument supports the technical review of guideline drafts.

#### 2.3.2 Delphi procedure

The items developed in Phase A were provided to the panel as the basis for rating. The Delphi thus began from a predefined item set rather than from open-ended idea generation (22). It was administered as a web-based questionnaire. Panelists rated their agreement with each item on a single 5-point scale (1=strongly disagree to 5=strongly agree), judging each item in terms of its validity, clarity, and clinical applicability; free-text fields allowed panelists to propose the revision, integration, deletion, or addition of items. Two rounds were planned: round 1 for rating of the items, and round 2, to be conducted only if consensus was not reached, in which panelists would receive controlled feedback and re-rate the items. The Delphi was to be terminated early if all items reached the consensus threshold in a round (23).

#### 2.3.3 Statistical analysis

Panel characteristics were summarized as frequencies and percentages. These comprised sex, age group, type of affiliation, prior involvement in KM-CPG planning, development, review, and/or accreditation, and area of expertise as a clinical expert and/or EBM methodologist; multiple responses were allowed for the involvement and expertise items. Percentages were calculated over the whole panel, except for years of clinical experience, which was summarized among clinical experts only.

The item-level content validity index (I-CVI) was computed for each item as the proportion of panelists rating it in the upper two categories (4–5); items so rated were treated as endorsed, following the dichotomization principle of the content validity index (24, 25). For each item, a chance-corrected modified kappa ($k^{*}$) was additionally calculated from the I-CVI and the binomial probability of chance agreement, with $k^{*}\geq 0.74$ conventionally regarded as excellent (26). The item-level I-CVI was additionally summarized by module. Consensus for an item was defined a priori as I-CVI≥0.80 (24). The module-system meta-evaluation item, which evaluates the module framework rather than an individual review point, was summarized separately by its I-CVI.

Three further indices, computed across the instrument’s items, were reported as supporting measures of agreement: the scale-level content validity index (S-CVI), Kendall’s coefficient of concordance (W), and the interquartile range (IQR). The S-CVI was calculated as the mean of the item-level I-CVIs (the S-CVI/Ave method), for which ≥0.90 is conventionally regarded as excellent content validity (25, 26). Kendall’s W (range 0–1) summarized the strength of agreement among panelists, with approximately 0.3, 0.5, and 0.7 taken to denote weak, moderate, and strong agreement (27). It was computed with mid-ranks assigned to ties and the corresponding tie correction applied. The IQR summarized response dispersion, with IQR≤1 on the 5-point scale commonly taken to indicate consensus (28).

The free-text comments were analyzed qualitatively by three researchers, who, through discussion, classified each comment and grouped the substantive suggestions by theme. All ratings were analyzed in aggregate across the full panel, without weighting by expert subgroup. Analyses were performed in R version 4.5.3 (R Foundation for Statistical Computing, Vienna, Austria). The figures were generated using Claude Code with the Claude Opus 4.8 model (Anthropic, San Francisco, CA, USA).

## 3 Results

### 3.1 Phase A: development of the draft instrument

#### 3.1.1 Derivation of the KM-EPRI draft

A total of 3,630 external peer-review comments were assembled, comprising all comments submitted by independent reviewers for 13 published KM-CPGs. Editorial and formatting comments, which made up a substantial share of this pool, were excluded (n=1,557), leaving 2,073 content-related comments (Supplementary Table S1). These comments were consolidated through a many-to-one mapping, in which multiple comments on the same review point were combined rather than discarded, producing a module-level draft instrument of 159 items (CQ, 7; CPG-I, 38; CPG-M, 39; CPG-R, 75). Additionally, at finalization, 42 items were removed as redundant or value-matching, that is, items overlapping with others and items that merely confirm that the same value appears consistently in different parts of a guideline (CPG-I, −7; CPG-R, −35). This produced the finalized draft instrument of 117 review items.

For the modified Delphi (Phase B), one module-system meta-evaluation item was then added to evaluate whether the CQ, CPG-I, CPG-M, and CPG-R module system is appropriately structured to review a guideline’s quality and clinical applicability from multiple perspectives. Because it addresses the module framework as a whole rather than any individual review point, it is not a component of the instrument. The Delphi assessment therefore comprised the 117 review items plus this single meta item (117+1; Figure 1).

**Figure 1:**
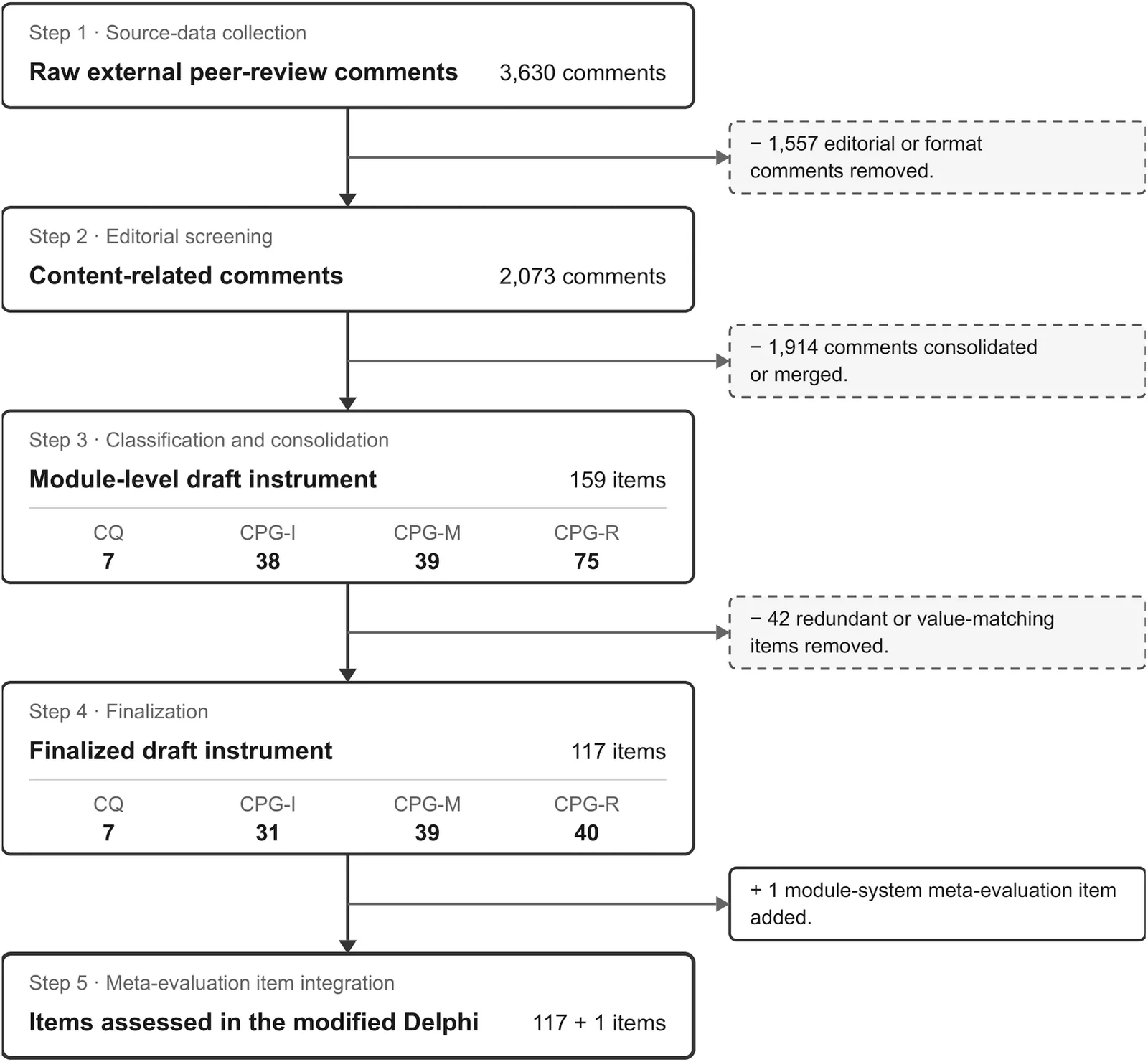
Development of the draft KM-EPRI. External peer review comments accumulated from 13 published KM-CPGs were processed across five steps. The unit of analysis shifts from individual comments (Steps 1–2) to review items (Steps 3–4). The finalized 117-item draft instrument was then carried into the modified Delphi, where it was assessed together with one module-system meta-evaluation item. CQ, clinical questions; CPG-I, clinical practice guideline-introduction; CPG-M, clinical practice guideline-methods; CPG-R, clinical practice guideline-recommendations.

#### 3.1.2 Structure of the KM-EPRI draft

The instrument is organized into 4 modules and 33 subdomains, whose labels follow the standardized section titles of the KM-CPG development manual (Ver 2.0) (11) (Figure 2). The CQ module (7 items across 5 subdomains) concerns the key clinical questions framed at the planning stage, addressing the patient population, the intervention and its comparator, the outcome measures, and the care setting. The CPG-I module (31 items, 9 subdomains) deals with the introductory content of a guideline: its background and scope, the definition and epidemiology of the disease, overviews of diagnosis and treatment, prevention and management, and plans for dissemination, implementation, monitoring, and updating. It also includes the clinical pathway diagram, a summary figure that reflects the guideline’s recommended diagnostic and treatment pathway. The CPG-M module (39 items, 11 subdomains) spans the development methodology, including the composition of the CDG, the handling of clinical questions, the search and selection of evidence, meta-analysis, and the assessment of the level of evidence and strength of recommendation. The CPG-R module (40 items, 8 subdomains) addresses the recommendations and their supporting evidence. It reviews the recommendation statements; the clinical considerations that translate each recommendation into practice by providing the specific information needed to apply it; the summaries of individual studies and meta-analyses; and the GRADE-based judgments expressed as the level of evidence and the strength of recommendation.

**Figure 2:**
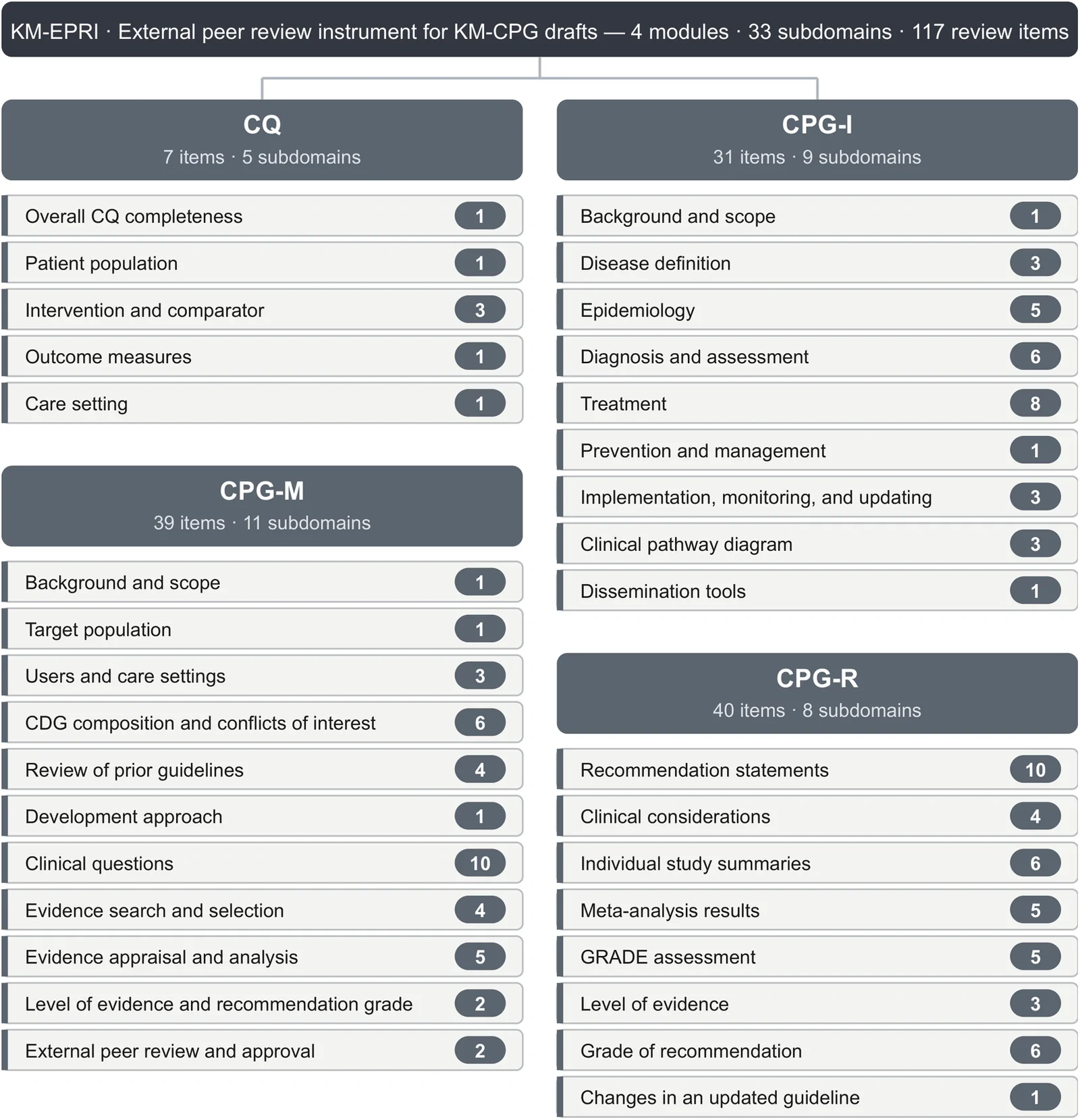
Structure of the finalized KM-EPRI. The finalized instrument comprises 117 review items grouped into 4 modules and 33 subdomains. Subdomain labels follow the standardized section titles defined in the KM-CPG development manual. The badge beside each subdomain gives the number of review items it contains. CQ, clinical questions; CPG-I, clinical practice guideline-introduction; CPG-M, clinical practice guideline-methods; CPG-R, clinical practice guideline-recommendations; CDG, clinical practice guideline development group; GRADE, Grading of Recommendations Assessment, Development and Evaluation.

### 3.2 Phase B: modified Delphi validation

#### 3.2.1 Characteristics of the expert panel in the modified Delphi

All 10 invited experts accepted and participated, for a response rate of 100% with no attrition. Although two rounds were planned, all items reached the consensus threshold in round 1, so the Delphi was completed in a single round under the pre-defined early-stopping rule. The panel was evenly split by sex and was predominantly in their 40s. Most panelists were affiliated with universities or university-affiliated hospitals (8/10); 8 were clinical experts and 6 were EBM methodologists, with 4 identifying as both. All 10 had previously served as KM-CPG reviewers, and most had also taken part in guideline development (8/10) or accreditation (9/10) (Table 1).

**Table 1:** Characteristics of the expert panel in the modified Delphi study (N=10).

Characteristic	n (%)
Sex	
Male	5 (50.0)
Female	5 (50.0)
Age group, years	
30–39	1 (10.0)
40–49	6 (60.0)
50–59	3 (30.0)
Affiliation	
University (including university-affiliated hospital)	8 (80.0)
National or public research institute	2 (20.0)
Prior involvement in KM-CPG processes${}^{a}$	
Planning	6 (60.0)
Development	8 (80.0)
Review	10 (100.0)
Accreditation	9 (90.0)
Area of expertise${}^{b}$	
Clinical expert	8 (80.0)
EBM methodologist	6 (60.0)
Clinical experience, years${}^{c}$	
5 to <10	1 (12.5)
10 to <20	5 (62.5)
≥20	2 (25.0)

Values are n (%).

KM-CPG, Korean Medicine clinical practice guideline; EBM, evidence-based medicine.

${}^{a}$Categories are not mutually exclusive. Panelists could report involvement in more than one process, so percentages do not sum to 100%.

${}^{b}$Categories are not mutually exclusive. Panelists could select both areas, and 4 panelists (40.0%) identified as both a clinical expert and an EBM methodologist.

${}^{c}$Clinical experience was queried only of the panelists who identified as clinical experts, so percentages are based on that subgroup (n=8).

#### 3.2.2 Content validity: I-CVI and S-CVI

Content-validity and agreement indices were computed for the 117 review items. The module-system meta-evaluation item differs in nature and is reported separately. All 117 items met the consensus threshold, with an I-CVI of at least 0.80. The distribution was 93 items (79.5%) at 1.00, 20 items (17.1%) at 0.90, and 4 items (3.4%) at 0.80; item-level indices are given in Supplementary Table S2. The S-CVI/Ave was 0.976, exceeding the 0.90 benchmark. After correction for chance agreement, all 117 items retained a modified kappa ($k^{*}$) of at least 0.79, within the range regarded as excellent (Supplementary Table S2). Mean I-CVI by module ranged from 0.943 to 0.990 (CQ, 0.943; CPG-I, 0.958; CPG-M, 0.990; CPG-R, 0.983), indicating consistent content validity across all modules (Table 2). The module-system meta-evaluation item received an I-CVI of 1.00, indicating that all panelists endorsed the appropriateness of the module framework.

**Table 2:** Modified Delphi results for the draft KM-EPRI, by review module.

Module	Items, n	I-CVI range	Mean I-CVI	Kendall’s W	Median IQR
CQ	7	0.80–1.00	0.943	0.156	0.75
CPG-I	31	0.80–1.00	0.958	0.204	0.75
CPG-M	39	0.90–1.00	0.990	0.184	0.00
CPG-R	40	0.90–1.00	0.983	0.150	0.00
**Overall**	**117**	**0.80–1.00**	**0.976**	**0.196**	**0.75**

Indices were computed over the 117 review items. The module-system meta-evaluation item is reported separately and is not included here.

KM-CPG, Korean Medicine clinical practice guideline; KM-EPRI, KM-CPG External Peer Review Instrument; CQ, clinical questions; CPG-I, clinical practice guideline–introduction; CPG-M, clinical practice guideline–methods; CPG-R, clinical practice guideline–recommendations; I-CVI, item-level content validity index; IQR, interquartile range.

Bold indicates the overall row, computed across all 117 review items.

#### 3.2.3 Rating agreement: Kendall’s W and IQR

The overall Kendall’s W was low, at 0.196, with module-level values ranging from 0.150 to 0.204 (CQ, 0.156; CPG-I, 0.204; CPG-M, 0.184; CPG-R, 0.150) (Table 2). Because W measures agreement on the relative ranking of items, it requires variation among items; the uniformly high ratings left little such variation, so these values reflect this restricted range rather than disagreement among panelists. The IQR was at most 1 for all 117 items (median 0.75; Supplementary Table S2), and the median rating of every item was at least 4.

#### 3.2.4 Panel suggestions in free-text comments

Panelists provided 22 free-text comments. Twelve were specific suggestions; the remaining 10 were set aside as 5 blank or “none” entries, 3 comments about the guideline accreditation process (which lies outside this study), and 2 brief notes of agreement.

The 12 suggestions addressed how interventions should be classified across the modules where conventional and KM overlap; which outcome measures to prefer; the consistency of pattern identification across source guidelines; the placement and synthesis of safety information; the coverage of certain review items, such as those for diagnostic and assessment tools; and where the boundary of the development scope should lie. Because all items had already met the consensus threshold, no item was revised, deleted, or re-circulated in response to these comments, which were instead recorded as qualitative feedback.

The finalized 117-item KM-EPRI is presented in full in Supplementary Table S3.

## 4 Discussion

### 4.1 Main findings: the instrument and its use

This study developed and validated KM-EPRI, an external peer review instrument for KM-CPG drafts. We derived the instrument from a large corpus of accumulated external review comments through directed qualitative content analysis, producing 117 review items across 4 modules and 33 subdomains. We then validated it through a modified Delphi study with a multidisciplinary panel of 10 experts who had served as KM-CPG reviewers. The panel reached consensus on all review items in a single round, and the results supported the content validity and acceptability of the instrument. KM-EPRI can give reviewers a structured, shared reference for reviewing guideline drafts during development, a function that post-hoc appraisal tools do not serve.

Because the items were derived from the comments that reviewers had actually raised, the instrument is weighted toward the problems that recur most often in practice. Aspects that reviewers frequently flag, such as imprecision and inconsistency in GRADE judgments, are represented in depth. Aspects that CDGs generally handle well, such as risk-of-bias assessment, drew fewer comments and are represented more lightly. KM-EPRI is a structured but non-scoring instrument. Each item names a point for the reviewer to consider and to address in free text, rather than a statement to be scored or agreed with. The items mark what a review should cover without limiting it to those points. Reviewers remain free to raise issues the instrument does not list.

### 4.2 Interpretation of the Delphi results

Consensus in Delphi studies is best judged with several measures rather than with any single coefficient (28). The I-CVI, the S-CVI/Ave, and the IQR pointed in the same direction across all four modules, indicating that the panel regarded the items as acceptable. The module-system meta-evaluation item, which was rated separately, was endorsed by all panelists. We read this as support for the four-module framework as an appropriate organizing structure, not as endorsement of any individual item.

The low Kendall’s W requires interpretation, because it appears to conflict with the high content-validity indices. W measures agreement on the relative ranking of items, so it requires variation among items to register that agreement (27). In this study the ratings were uniformly high, with most items at the top of the scale and an IQR of at most 1. This ceiling effect left little variation among items for W to detect. The coefficient came out low even though absolute agreement among panelists was strong (29).

The Delphi was completed in a single round. Under the pre-defined early-stopping rule, every item met the consensus threshold in round 1, so a second round was not warranted (23). A second round in a Delphi serves to feed back the distribution of first-round responses and let panelists reconsider items on which the group diverged. With consensus already reached on every item, no such divergence remained for a further round to resolve. Early stopping was a planned feature of the design rather than a shortcut. It does mean that the panel did not pass through a controlled feedback-and-rerating cycle, and we return to this point as a limitation.

### 4.3 Comparison with appraisal tools and review practices

KM-EPRI is related to AGREE II but occupies a different position in the guideline life cycle. AGREE II appraises the overall quality of a completed guideline, and it treats external review as a single confirmatory item (13). KM-EPRI is instead intended to structure the content of a section-by-section review of a draft. Both examine how the evidence was searched, selected, and appraised, and how the recommendations follow from it under GRADE. Both also address the composition of the CDG, the handling of conflicts of interest, the clarity of the recommendation statements, and the plan for updating. The two instruments differ in the level at which they operate and in what they cover. AGREE II is applied to a guideline as a whole, whereas KM-EPRI works through it section by section, examining each recommendation together with its supporting studies and its GRADE judgment. KM-EPRI also covers two areas that AGREE II leaves out: the clinical content of the guideline, such as the definition, epidemiology, diagnosis, and treatment of the disease, and the clinical questions framed at the planning stage.

KM-EPRI also differs from the external review practices of national and international guideline organizations. These organizations standardize the process of review, specifying who reviews, when review occurs, and how comments are documented (1–7, 30). The content of the review, however, is generally left to open-ended comment or general consultation rather than to a structured, item-level instrument. Reviewers are asked for reasoned argument, for judgments on the comprehensiveness and accuracy of the evidence, or on the appropriateness of recommendations, but not to work through components mapped to the sections of a draft. To our knowledge, none of these organizations provides reviewers with an item-level review instrument tied to the structure of a guideline draft. KM-EPRI is designed to fill the gap that these process frameworks leave open, namely the content of the review.

### 4.4 Strengths and limitations

This study has several strengths. The instrument was derived empirically from a large body of real external review comments, so its items reflect the issues that arise in actual guideline review. Its modular design lets reviewers apply the relevant module to the section under review. Although the modules were built from the KM-CPG development manual, much of their content concerns elements common to guidelines in general, such as the framing of clinical questions, the appraisal of evidence, and the formulation of recommendations. KM-EPRI could therefore serve as a prototype for review instruments in guideline systems beyond KM.

Several limitations should be noted. First, although 10 experts are considered an adequate panel size for content validity (24), the panel was small, and its eligibility criteria were broad. Panel recruitment was closed once 10 experts were enrolled, and eligibility required only one of guideline involvement, methodological expertise, or clinical experience. The panel’s endorsement of the module framework may reflect familiarity as much as appropriateness. Relatedly, the items were derived from external review comments and the panel was drawn from the same expert community, so development and validation were not fully independent. Because KM-EPRI is a reference rather than a scoring tool, the panel might also have had little incentive for the sharp discrimination that produces rank variance, consistent with the ceiling effect discussed above. Second, KM-EPRI has not yet been applied in actual review, so its effect on inter-rater reliability, its feasibility, and its contribution to the quality of review remain unmeasured. This leaves the instrument’s central aims untested, namely improving inter-rater reliability and aligning the expectations of developers and reviewers (14, 15). Third, because the items were derived empirically, those raised infrequently in past reviews are under-represented. We sought to offset this in the instrument’s instructions, which tell reviewers that the items are not exhaustive and that other relevant issues should still be raised. Fourth, KM-EPRI was developed within a single national KM context, which limits its generalizability, and validated through a single Delphi round, which did not include a controlled feedback cycle. Fifth, the instrument contains 117 items, which would be a substantial workload for a reviewer. In practice, the modules can be divided among reviewers so that each works with a part of the draft. In the KM-CPG setting, 12 to 16 external reviewers are assigned to the review of a guideline (Supplementary Table S1). However, it may be necessary to condense the items or apply them selectively when a single reviewer covers an entire draft.

### 4.5 Implications for practice and future research

KM-EPRI follows the section structure of the KM-CPG development manual and includes items specific to KM. Therefore, applying the instrument directly to other guideline systems may be difficult. However, its four modules can be suitable for any guideline developed under Cochrane methods and GRADE. Within each module, the specific review items can be modified to align with each guideline system. This would be particularly useful in traditional-medicine guidelines, where pattern identification or recommendations for combined or adjunctive therapies alongside conventional care are usually included.

A shared instrument should make review more consistent and give new reviewers a concrete training reference. It can also be used in part, so a reviewer responsible for a single section can work from the corresponding module alone. Delivering the instrument in digital form would allow reviewers to record comments against each item and enable collation by module.

KM-EPRI does not cover how review comments are published and resolved, or whether a draft is opened to public comment. How the instrument and these procedural elements should work together is one direction for future work. Another is to test the instrument in use, examining its effect on inter-rater reliability and its feasibility. Beyond the content validity assessed here, construct and criterion validity also remain to be tested. The revision of the instrument over time, and its transfer to other traditional-medicine and conventional guideline systems, are also worth studying. Subject to this further validation, KM-EPRI offers a consensus-based foundation for standardizing the external peer review of KM-CPG drafts.

## Data Availability

The raw data supporting the conclusions of this article will be made available by the authors, without undue reservation.

## Supplementary material

Supplementary Table S1 Overview of the source guidelines included in KM-EPRI draft development.

Supplementary Table S2 Item-level Delphi results for the draft KM-EPRI.

Supplementary Table S3 The finalized KM-EPRI instrument.
