# Supplementary material for: An external peer review instrument for Korean Medicine clinical practice guideline drafts (KM-EPRI): development and content validation
Source: Front Med (Lausanne). 2026 Sep 23;13:1926917. doi: 10.3389/fmed.2026.1926917 (PMC13642740; doi:10.3389/fmed.2026.1926917)
Supplement: Supplementary file 1 [file Table1.pdf]

Supplementary Material

The following supplementary tables accompany the article. Each table is preceded by its caption; notes and abbreviations follow each table.

Supplementary Table S1. Overview of the source guidelines included in KM-EPRI draft development.

#	Guideline	Publication date	ISBN	Unique reviewers (n)	Review rounds	Total comments (n)	Editorial or format comments (n)	Content-related comments (n)
1	Smoking cessation	2023-06-12	979-11-93073-02-5	14	2	234	84	150
2	Obesity	2024-04-12	979-11-93073-08-7	14	3	380	153	227
3	Pediatric anorexia	2024-04-12	979-11-93073-07-0	16	3	305	37	268
4	Postpartum wind disorder (Sanhupung)	2024-04-12	979-11-93073-05-6	16	3	228	77	151
5	Premenstrual syndrome	2024-04-24	979-11-93073-04-9	13	2	193	83	110
6	Female infertility	2024-04-25	979-11-93073-10-0	15	1	349	228	121
7	Depression	2024-05-17	979-11-93073-06-3	16	2	524	289	235
8	Nausea and vomiting of pregnancy	2024-05-17	979-11-93073-12-4	14	2	238	107	131
9	Osteoarthritis (hip and hand)	2024-05-17	979-11-93073-11-7	12	3	142	62	80
10	Rheumatoid arthritis	2024-05-31	979-11-93073-09-4	12	3	198	101	97
11	Autonomic dysfunction	2024-10-25	979-11-93073-13-1	13	2	314	108	206
12	Benign prostatic hyperplasia	2024-10-25	979-11-93073-14-8	13	2	176	68	108
13	Fracture	2025-03-14	979-11-93073-16-2	14	3	349	160	189
	**Mean**			**14.0**	**2.4**	**279.2**	**119.8**	**159.5**

#	Guideline	Publication date	ISBN	Unique reviewers (n)	Review rounds	Total comments (n)	Editorial or format comments (n)	Content-related comments (n)
	**Median**			**14**	**2**	**238**	**101**	**150**
	**Range**			**12–16**	**1–3**	**142–524**	**37–289**	**80–268**

The source corpus comprised all external peer-review comments from the 13 KM-CPGs developed under the KM-CPG development manual, version 2.0, and published by August 2025. Unique reviewers are the distinct individuals per guideline, each counted once. Total comments are all external peer-review comments collected for a guideline. Editorial or format comments are the subset removed at editorial screening (Phase A, Step 2). Content-related comments (total comments minus editorial or format comments) were carried into Phase A Step 3.

As an example of how to read a row, the Depression guideline received 524 comments from 16 reviewers over two review rounds during its development; 289 of these were editorial or format comments, so the remaining 235 content-related comments were used in developing the draft KM-EPRI.

Abbreviations: ISBN, International Standard Book Number; KM-EPRI, KM-CPG External Peer Review Instrument; KM-CPG, Korean Medicine clinical practice guideline; n, number.

Supplementary Table S2. Item-level Delphi results for the draft KM-EPRI.

Module	ID	I-CVI	k*	Md	IQR
CQ	01	1.00	1.000	5.0	0.75
	02	1.00	1.000	5.0	0.75
	03	1.00	1.000	5.0	0.75
	04	1.00	1.000	4.5	1.00
	05	0.80	0.791	4.0	0.75
	06	0.90	0.899	5.0	0.75
	07	0.90	0.899	4.0	1.00
CPG-I	01	0.80	0.791	5.0	1.00
	02	1.00	1.000	5.0	0.00
	03	1.00	1.000	5.0	0.75
	04	1.00	1.000	5.0	1.00
	05	1.00	1.000	5.0	1.00
	06	1.00	1.000	5.0	0.75
	07	1.00	1.000	5.0	0.75
	08	1.00	1.000	5.0	0.75
	09	1.00	1.000	5.0	1.00
	10	1.00	1.000	5.0	0.00
	11	1.00	1.000	5.0	1.00
	12	1.00	1.000	5.0	1.00
	13	1.00	1.000	4.5	1.00
	14	1.00	1.000	4.0	1.00
	15	0.80	0.791	4.0	0.75
	16	1.00	1.000	5.0	0.00
	17	1.00	1.000	5.0	0.75
	18	0.80	0.791	4.0	1.00
	19	1.00	1.000	4.0	1.00
	20	0.90	0.899	5.0	1.00
	21	0.90	0.899	5.0	1.00
	22	0.90	0.899	5.0	1.00
	23	1.00	1.000	5.0	0.00
	24	1.00	1.000	4.0	0.00
	25	0.90	0.899	5.0	0.75
	26	0.90	0.899	5.0	0.75
	27	0.90	0.899	5.0	0.00
	28	1.00	1.000	5.0	0.00
	29	0.90	0.899	5.0	0.00
	30	1.00	1.000	5.0	0.00
	31	1.00	1.000	5.0	0.75
CPG-M	01	1.00	1.000	5.0	0.75
	02	1.00	1.000	5.0	0.00
	03	1.00	1.000	4.0	1.00
	04	1.00	1.000	5.0	0.00
	05	1.00	1.000	5.0	0.75
	06	1.00	1.000	5.0	0.75
	07	1.00	1.000	5.0	0.00
	08	1.00	1.000	5.0	1.00
	09	1.00	1.000	5.0	0.00
	10	1.00	1.000	5.0	0.00
	11	1.00	1.000	5.0	1.00
	12	1.00	1.000	5.0	0.00
	13	1.00	1.000	5.0	0.75
	14	1.00	1.000	5.0	1.00
	15	1.00	1.000	5.0	0.00
	16	1.00	1.000	5.0	0.00
	17	1.00	1.000	5.0	0.00
	18	0.90	0.899	5.0	1.00
	19	1.00	1.000	5.0	0.75
	20	1.00	1.000	4.5	1.00
	21	1.00	1.000	5.0	1.00
	22	1.00	1.000	5.0	1.00
	23	1.00	1.000	5.0	0.75
	24	0.90	0.899	4.5	1.00
	25	1.00	1.000	5.0	0.75
	26	1.00	1.000	5.0	1.00
	27	1.00	1.000	5.0	0.00
	28	1.00	1.000	5.0	0.00
	29	1.00	1.000	5.0	0.00
	30	0.90	0.899	5.0	0.75
	31	1.00	1.000	5.0	0.00
	32	1.00	1.000	5.0	0.00
	33	1.00	1.000	5.0	0.00
	34	1.00	1.000	5.0	0.00
	35	1.00	1.000	5.0	0.00
	36	1.00	1.000	5.0	0.00
	37	1.00	1.000	5.0	0.00
	38	0.90	0.899	5.0	1.00
	39	1.00	1.000	5.0	0.00
CPG-R	01	1.00	1.000	5.0	0.75
	02	1.00	1.000	5.0	0.75
	03	1.00	1.000	5.0	0.00
	04	1.00	1.000	4.5	1.00
	05	1.00	1.000	5.0	0.00
	06	1.00	1.000	5.0	1.00
	07	1.00	1.000	4.0	1.00
	08	0.90	0.899	5.0	1.00
	09	1.00	1.000	5.0	0.75
	10	1.00	1.000	5.0	1.00
	11	1.00	1.000	5.0	0.00
	12	1.00	1.000	5.0	0.00
	13	1.00	1.000	5.0	0.00
	14	0.90	0.899	5.0	0.00
	15	0.90	0.899	5.0	0.00
	16	1.00	1.000	5.0	0.75
	17	1.00	1.000	5.0	0.75
	18	1.00	1.000	5.0	0.00
	19	1.00	1.000	5.0	0.00
	20	1.00	1.000	5.0	0.00
	21	1.00	1.000	5.0	0.00
	22	1.00	1.000	5.0	0.00
	23	1.00	1.000	5.0	0.00
	24	1.00	1.000	5.0	0.00
	25	0.90	0.899	5.0	0.75
	26	1.00	1.000	5.0	0.00
	27	1.00	1.000	5.0	0.00
	28	1.00	1.000	5.0	0.00
	29	1.00	1.000	5.0	0.00
	30	0.90	0.899	5.0	0.75
	31	1.00	1.000	5.0	0.00
	32	1.00	1.000	5.0	0.00
	33	1.00	1.000	5.0	0.00
	34	1.00	1.000	5.0	0.00
	35	1.00	1.000	5.0	0.00
	36	1.00	1.000	5.0	0.00
	37	0.90	0.899	5.0	0.75
	38	0.90	0.899	5.0	1.00
	39	1.00	1.000	5.0	0.00
	40	1.00	1.000	5.0	0.75

I-CVI, k*, median, and IQR were derived from the 10 panelists' ratings on a 5-point agreement scale (1 = strongly disagree to 5 = strongly agree). k* is the chance-corrected modified kappa for the I-CVI, computed as $k^* = (\text{I-CVI} - Pc)/(1 - Pc)$, where Pc is the binomial probability of chance agreement; $k^* \geq 0.74$ is regarded as excellent.

Abbreviations: CPG-I, clinical practice guideline–introduction; CPG-M, clinical practice guideline–methods; CPG-R, clinical practice guideline–recommendations; CQ, clinical questions; I-CVI, item-level content validity index; ID, item identifier; IQR, interquartile range; k*, chance-corrected modified kappa; KM-EPRI, KM-CPG External Peer Review Instrument; KM-CPG, Korean Medicine clinical practice guideline; Md, median.

Supplementary Table S3. The finalized KM-EPRI instrument.

External Peer Review Instrument for Drafts of Korean Medicine Clinical Practice Guidelines (KM-EPRI)

The KM-EPRI is an instrument to support external peer review of KM-CPG drafts during development, as distinct from the post-hoc appraisal of completed guidelines such as AGREE II. It comprises 117 review items organized into four technical-review modules (CQ, CPG-I, CPG-M, and CPG-R), which mirror the main parts of a KM-CPG: the clinical questions, the introduction, the methods, and the recommendations. Within each module, the items are grouped into subdomains that follow the section structure prescribed by the KM-CPG development manual, so that each item maps to the corresponding part of the draft. In use, the modules can be divided among reviewers so that each reviewer works through the assigned module, recording specific comments and revision suggestions where the draft falls short.

The items were derived from comments that reviewers had actually raised, so the instrument is weighted toward the problems that recur most often in practice. KM-EPRI is an open-ended instrument, not a rating scale. Each item names a point for the reviewer to consider and to address in free text, rather than a statement to be scored or agreed with. The items mark what a review should cover but do not limit it, and reviewers remain free to raise issues the instrument does not list.

Subdomain	ID	Review item
**Module: CQ**		
**Overall CQ completeness**	01	Review whether the CQs include all PICO elements.
**Patient population**	02	Review whether the patient population is defined at a clinically appropriate level of detail: narrowed by relevant characteristics (age, severity, pattern identification) where too broad, or consolidated with similar populations where too narrow.
**Intervention and comparator**	03	Review whether the intervention and comparator are defined specifically: specify the sub-type of a broad term (e.g., manual or electroacupuncture instead of just acupuncture), and for a composite comparator, list its component treatment modalities.
	04	Review whether the CQs cover all interventions and comparators in use or applicable in clinical practice.
	05	Review whether interventions and comparators are classified as KM or conventional (e.g., standard conventional drug therapy instead of just standard drug therapy), and whether the same intervention is classified consistently across CQs.
**Outcome measures**	06	Review whether the outcome measures are specific (e.g., pain or degree of depression instead of

Subdomain	ID	Review item
		symptom or effect).
**Care setting**	07	Review whether the care setting is fully defined and applicable in clinical practice; for a CQ on KM–conventional collaborative care, specify the hospital setting in which it can be delivered.
**Module: CPG-I**		
**Background and scope**	01	Review whether the development background adequately covers what the recommendations address, such as the patient conditions they specify (e.g., pain of VAS ≥ 4, post-surgical low back pain, etc.) and the KM interventions they include.
**Disease definition**	02	Review whether the target disease is defined appropriately, with sound terminology; where the disease concept is separately defined, assess the validity of that definition.
	03	Review whether the definition of the target disease aligns with the recommendations, reflecting any severity or prior-treatment criteria used to specify the target patients.
	04	Review whether the KM characterization of the target disease (e.g., etiology, pathomechanism, pattern identification, etc.) is appropriate.
**Epidemiology**	05	Review whether the epidemiology aligns with the recommendations, reflecting any severity or prior-treatment criteria used to specify the target patients.
	06	Review whether the epidemiological statistics are reported with their terms, computed values, units, reference dates, and sources.
	07	Review whether the epidemiological statistics draw on the most recent data.
	08	Review whether the epidemiological statistics are interpreted accurately and within their actual scope.
	09	Review whether KM-specific epidemiology and practice patterns are included (e.g., surveys of KM doctors, surveys of patients at KM institutions, practice status from claims data, etc.).
**Diagnosis and assessment**	10	Review whether diagnosis uses current criteria

Subdomain	ID	Review item
		applicable in clinical practice, with differential diagnosis covered where relevant.
	11	Review whether the diagnostic criteria align with the recommendations (e.g., reflecting any limited severity range the recommendation targets).
	12	Review whether diagnostic and assessment tools are described in enough detail for use in clinical practice, including the tool's name, scoring and interpretation, reliability and validity, official translation, and any copyright or cost issues.
	13	Review whether the diagnostic and assessment tools align with the recommendations.
	14	Review whether KM pattern identification is described appropriately, including pattern types, criteria, methods, and tools.
	15	Review whether the KM pattern identification aligns with the recommendations.
**Treatment**	16	Review whether the treatment modalities applicable in clinical practice are described in detail.
	17	Review whether the treatment modalities reflect the latest developments in the field.
	18	Review whether usual care, standard care, and the like are classified as KM or conventional.
	19	Review whether usual care, standard care, and the like, whether KM or conventional, are described in detail.
	20	Review whether treatment modalities that KM doctors can legally and customarily perform are classified as KM where applicable.
	21	Review whether KM treatment modalities are described in detail (e.g., herbal medicine: formula name, herbs, pattern, treatment principle, modifications, etc.; acupoint-based treatment such as acupuncture and moxibustion: acupoints, pattern, treatment principle, modifications, etc.; manual and mind-body therapies: procedures, contraindications, precautions, etc.).

Subdomain	ID	Review item
	22	Review whether safety information for KM treatment modalities is described, especially for vulnerable groups such as children, older adults, pregnant women, etc.
	23	Review whether the treatment modalities align consistently with the interventions and comparators in the recommendations.
**Prevention and management**	24	Review whether prevention and management are described from KM and/or conventional perspectives.
**Implementation, monitoring, and updating**	25	Review whether the guideline gives specific strategies for use by patients, practitioners, learners (KM students, trainees, and continuing-education participants), and policymakers.
	26	Review whether the guideline provides specific plans and methods to evaluate its implementation and performance.
	27	Review whether the updating plan specifies who updates the guideline, how often, under what conditions, and by what procedure.
**Clinical pathway diagram**	28	Review whether the diagnostic process is reflected in the clinical pathway diagram of the guideline.
	29	Review whether the recommended treatment modalities, with their level of evidence and grade of recommendation, are accurately reflected in the clinical pathway diagram of the guideline.
	30	Review whether the clinical pathway diagram is easy to follow in clinical practice.
**Dissemination tools**	31	Review whether dissemination tools such as leaflets, infographics, etc., are useful to both practitioners and patients in clinical practice.
**Module: CPG-M**		
**Background and scope**	01	Review whether the rationale for developing a KM-based guideline is explained.
**Target population**	02	Review whether the target disease aligns with the recommendations, reflecting any severity or prior-treatment criteria used to specify the target patients.
**Users and care settings**	03	Review whether the users, such as clinical KM

Subdomain	ID	Review item
		doctors, other health professionals, learners (KM students, trainees, and continuing-education participants), policymakers, etc., are appropriately identified.
	04	Review whether the guideline considers the full range of care settings where KM is delivered (e.g., KM clinics, KM hospitals, long-term care hospitals, public health centers, etc.).
	05	Review whether the guideline describes users and care settings specifically enough to reflect real-world practice (e.g., institutions offering KM–conventional collaborative care, public health KM doctors, etc.).
**CDG composition and conflicts of interest**	06	Review whether each CDG member's affiliation, region, specialty, and role is specified.
	07	Review whether the CDG includes both clinical and methodology experts.
	08	Review whether the CDG is diverse in affiliation, region, and specialty.
	09	Review whether each committee's role is defined and the committee involved in the final consensus on the recommendations is specified, if the CDG is organized into several committees.
	10	Review whether all CDG members declared conflicts of interest transparently.
	11	Review whether specific safeguards (e.g., independent third-party review, dual review, methodologist verification, excluding the study, etc.) minimize the risk of bias when a CDG member's own study serves as evidence.
**Review of prior guidelines**	12	Review whether prior Korean and overseas guidelines were comprehensively examined.
	13	Review whether the information identified on prior Korean and overseas guidelines is reported in detail (e.g., title, issuing body, year, key recommendations, etc.).
	14	Review whether developing a new guideline is justified by prior Korean and overseas guidelines'

Subdomain	ID	Review item
		inapplicability to KM practice.
	15	Review whether updating the guideline is justified by the previous guideline's limitations.
**Development approach**	16	Review whether the development approach is specified as de novo, adaptation, or hybrid.
**Clinical questions**	17	Review whether the CQs are structured by PICO and presented in a user-friendly form.
	18	Review whether the CQs are framed for clinical use rather than as units of analysis (e.g., negative controls such as placebo, no treatment, etc., may be combined into a single CQ even if the quantitative analysis is conducted separately).
	19	Review whether terminology is used consistently across CQs (e.g., disease labels, intervention classifications as KM or conventional, intervention names, etc.).
	20	Review whether each CQ's population is defined at a clinically appropriate level of detail: narrowed by relevant characteristics (age, severity, pattern identification) where too broad, or consolidated with similar populations where too narrow.
	21	Review whether interventions are classified as KM monotherapy, KM combination, or KM–conventional combination.
	22	Review whether the CQs' interventions and comparators are defined specifically, and whether usual care, standard care, and the like are classified as KM or conventional with their component treatment modalities specified.
	23	Review whether outcome measures are defined specifically, with assessment tools and methods where needed.
	24	Review whether each CQ's care setting (e.g., KM clinic, KM hospital, etc.) is fully defined and appropriate for delivering the relevant intervention.
	25	Review whether the CQs cover all relevant practices in use or applicable in clinical practice.

Subdomain	ID	Review item
	26	Review whether changes (additions, deletions, merges, edits) to the CQs during development are documented with reasons.
**Evidence search and selection**	27	Review whether the search databases, platform, and provider are specified accurately.
	28	Review whether the search is up to date and whether a top-up search is needed, given the time span between the last search and review.
	29	Review whether the search terms are well chosen and reported, including controlled vocabulary per database (e.g., MeSH, Emtree, etc.) and key terms per language (e.g., Korean, English, Chinese, Japanese, etc.).
	30	Review whether excluding observational studies is justified when only RCTs are included, and whether including them was considered where RCTs alone may be insufficient, such as for safety.
**Evidence appraisal and analysis**	31	Review whether the risk-of-bias tool (e.g., RoB 2.0, ROBINS, etc.) is specified accurately.
	32	Review whether the outcomes in the meta-analysis are specified and match those used for the recommendations.
	33	Review whether the choice of effect estimate (e.g., MD/SMD, OR/RR/RD, etc.) is justified.
	34	Review whether the choice between fixed-effect and random-effects models is justified.
	35	Review whether the methods and thresholds for judging heterogeneity (e.g., I^2, Q statistic, etc.) are specified.
**Level of evidence and recommendation grade**	36	Review whether the definitions and criteria for level of evidence and grade of recommendation follow the latest guideline development manuals.
	37	Review whether the consensus method (e.g., Delphi, etc.) is specified with its participants, items, criteria, etc., for consensus-based recommendations.
**External peer review and approval**	38	Review whether the external peer reviewers' identities, expertise, and roles are described, and

Subdomain	ID	Review item
		whether CDG members were excluded from external peer review.
	39	Review whether each academic society that approved the guideline is described individually with its name, approval date, and approval procedure.
**Module: CPG-R**		
**Recommendation statements**	01	Review whether every CQ has a recommendation and none is omitted; where no conclusion can be reached, an 'Inconclusive' recommendation should be issued.
	02	Review whether the recommendation accurately reflects all of the CQ's PICO elements.
	03	Review whether the recommendation is unambiguous and clinically distinguishable from similar recommendations, and describes each PICO element with consistent criteria.
	04	Review whether recommendations are split or combined based on clinical differences and framed as KM monotherapy, KM combination, or KM–conventional combination.
	05	Review whether the recommendation states the patient's clinical features and the intervention in standard terms and is consistent with the practical details in the Clinical Considerations section.
	06	Review whether the treatment goal conveys clinical meaning rather than merely an outcome measure's name (e.g., reduced depression instead of a lower BDI score).
	07	Review whether the recommendation stays within what the included studies support (e.g., it is inappropriate to recommend acupuncture broadly when only electroacupuncture was studied).
	08	Review whether the recommendation avoids including the comparator unnecessarily for CQs that set a negative control (placebo, sham acupuncture, no treatment, etc.).
	09	Review whether a sub-recommendation is justified

Subdomain	ID	Review item
		and its level of evidence and grade were assessed independently of the parent recommendation, if any.
	10	Review whether a diagnostic recommendation, if any, supports clinical decision-making rather than merely restating usual diagnostic criteria.
**Clinical considerations**	11	Review whether the Clinical Considerations section is specific and clinician-friendly, e.g., herbal medicine (name; herbs and doses; administration; frequency; duration; precautions and contraindications; modification rules; etc.); acupoint-based treatment (technique such as manual, auricular, or electroacupuncture and direct or indirect moxibustion; acupoints; stimulation time and intensity; frequency; duration; etc.); pharmacopuncture (solution type, concentration, volume, frequency, duration, precautions and contraindications, etc.); and Chuna, mind-body, exercise therapy, and the like (procedure, sessions, duration, precautions and contraindications, etc.).
	12	Review whether the Clinical Considerations section describes usual care, standard care, and the like in detail when the recommendation uses such terms.
	13	Review whether expected adverse events, precautions, and contraindications are presented in the Clinical Considerations section where safety concerns may arise.
	14	Review whether any clinical difference (indications, patient condition, etc.) is specified in the Clinical Considerations section when the same intervention is recommended both alone and in combination.
**Individual study summaries**	15	Review whether individual study summaries give design, sample size, and specific intervention and control detail, with no need to list every outcome value and its significance.
	16	Review whether a multi-arm study's summary identifies which arms were actually used for the recommendation.
	17	Review whether adverse events are reported by

Subdomain	ID	Review item
		group for individual studies, and whether their absence is explicitly noted where none occurred.
	18	Review whether individual studies' interventions and comparators are applicable in clinical practice, with a clear rationale where uncommon or rarely used treatment modalities are cited as evidence.
	19	Review whether individual studies' PICO matches the CQ, with a rationale where it does not.
	20	Review whether, for consensus-based recommendations, the guideline summarizes the related studies (non-randomized and observational, etc.) and states the reason for adopting consensus.
**Meta-analysis results**	21	Review whether outcomes are pooled or separated considering clinical, not just statistical, heterogeneity in the meta-analysis.
	22	Review whether pooling reflects each control's nature and clinical availability in the meta-analysis: negative controls (e.g., no treatment, placebo, etc.) should be pooled, while active controls can be pooled or separated by their clinical similarity.
	23	Review whether the meta-analysis estimates (e.g., MD, SMD, RR, OR, RD, etc.) fit the outcome type.
	24	Review whether statistical adjustments are applied to prevent overestimation of the effect size when an included study contributes multiple time points or arms in the meta-analysis.
	25	Review whether adverse events are meta-analyzed, without misreading fewer adverse events as efficacy or no adverse events as proof of safety.
**GRADE assessment**	26	Review whether all GRADE domains are rated, with reasons given per outcome.
	27	Review whether GRADE domains are rated consistently across recommendations.
	28	Review whether the GRADE ratings are appropriately reflected in each outcome's level of evidence, including downgrading where a domain is rated as serious.

Subdomain	ID	Review item
	29	Review whether the inconsistency rating weighs both the size of heterogeneity and the consistency of effect direction across included studies.
	30	Review whether the imprecision rating weighs the CI's overlap with no effect, its width, and the OIS threshold.
**Level of evidence**	31	Review whether the importance of each outcome used to derive the overall level of evidence is given.
	32	Review whether the overall level of evidence is linked to the individual outcomes' levels of evidence and set conservatively at the lowest among the critical outcomes, with reasons for any up- or downgrading.
	33	Review whether no level of evidence is assigned to consensus-based recommendations, which lack quantitative analysis.
**Grade of recommendation**	34	Review whether the grade of recommendation is justified across benefits and harms, overall level of evidence, clinical availability, cost-effectiveness, and values and preferences.
	35	Review whether the level of evidence and grade of recommendation are linked, with reasons for any up- or downgrading (e.g., the methods and results of preference or utilization surveys when upgrading for clinical availability; the consensus process and results when upgrading for expert consensus; etc.).
	36	Review whether the overall benefit is judged appropriately, including specific reasons for recommending clinical use despite non-significant meta-analysis results.
	37	Review whether the overall harm is judged comprehensively from adverse-event meta-analyses, prior reports, expert opinion, etc.
	38	Review whether cost-effectiveness is judged appropriately; where no economic evaluation is available, the judgment may rest on expert opinion based on factors such as insurance coverage, etc.
	39	Review whether, for consensus-based

Subdomain	ID	Review item
		recommendations, the consensus process and results are described, with any minority opinions summarized.
**Changes in an updated guideline**	40	Review whether an updated guideline describes the changes from the previous version (e.g., new or revised recommendations, added or removed evidence, changes in level of evidence or grade of recommendation, etc.).

Abbreviations: AGREE II, Appraisal of Guidelines for Research and Evaluation II; BDI, Beck Depression Inventory; CDG, clinical practice guideline development group; CI, confidence interval; CPG-I, clinical practice guideline–introduction; CPG-M, clinical practice guideline–methods; CPG-R, clinical practice guideline–recommendations; CQ, clinical questions; GRADE, Grading of Recommendations Assessment, Development and Evaluation; ID, item identifier; KM-EPRI, KM-CPG External Peer Review Instrument; KM, Korean medicine; KM-CPG, Korean Medicine clinical practice guideline; MD, mean difference; OIS, optimal information size; OR, odds ratio; PICO, population, intervention, comparison, outcome; RCT, randomized controlled trial; RD, risk difference; RR, risk ratio; SMD, standardized mean difference; VAS, visual analog scale.
